# Endothelial MerTK impairment promotes cardiac dysfunction in the condition of high fat diet

**DOI:** 10.1016/j.redox.2026.104184

**Published:** 2026-04-21

**Authors:** Hongye Huang, Shijie Liu, Jingke Yao, Xiaoyuan Bai, Zhicheng Jin, Bingzhong Xue, Hang Shi, Zufeng Ding

**Affiliations:** a Department of Biology, Georgia State University, Atlanta, GA, 30303, USA; b Department of Chemistry, Georgia State University, Atlanta, GA, 30303, USA

**Keywords:** MerTK, Endothelial cells, Cardiac dysfunction, Big data analytics, scRNA-seq

## Abstract

**Rationale:**

Cardiac fibrosis formation leads to cardiac dysfunction that is mainly caused by a high fat diet accompanied by an increased accumulation of apoptotic cells. MER proto-oncogene tyrosine kinase (MerTK) is a major receptor for efferocytosis, a process for the efficient clearance of apoptotic cells. This study was designed to investigate the novel role of endothelial MerTK in regulating cardiac dysfunction in the condition of high fat diet.

**Methods:**

MerTK deficiency in endothelial cells (*MerTK*^*flox/flox*^*Tie2*^*Cre*^) mice and the littermates of *MerTK*^*flox/flox*^ mice were injected with a single dose of AAV8-PCSK9 particles, followed by a high fat diet for two months. Multi-omics approach includes big data analytics and proteomics as well as single-cell RNA sequencing (scRNA-seq) and single-nucleus RNA sequencing (snRNA-seq) with human specimens. Immunostaining validation in vivo were also utilized to elucidate the underlying mechanisms of endothelial MerTK in cardiac dysfunction.

**Results:**

The proteomics data showed that mitochondrial dysfunction, increased apoptosis and necrosis, defective phagosome formation, and impaired engulfment of cells represent the main signaling pathways involved in endothelial MerTK-mediated cardiac dysfunction. The immunostaining data indicates that endothelial MerTK deficiency promotes NADPH oxidases activation, cardiac fibrosis formation, phenotypic switching in smooth muscle cells (SMCs) and pro-inflammation response, while inhibiting expression of Apolipoprotein E (ApoE), all are key drivers to accelerate cardiac dysfunction. The scRNA-seq analysis in mouse hearts highlights the importance of endothelial functions in cardiac hypertrophy. The snRNA-seq analysis reveals endothelial MerTK dynamics in cardiac dysfunction based on specimens from human patients with dilated cardiomyopathy (DCM) and hypertrophic cardiomyopathy (HCM).

**Conclusions:**

These findings provide compelling evidence that endothelial MerTK impairment is a novel mechanism in promoting cardiac dysfunction.

## Highlights

• The role of endothelial MerTK in cardiac function remains unknown.
• Multi-omics were performed to cross-validate the role of endothelial MerTK in cardiac dysfunction.
• Endothelial MerTK deficiency promotes cardiac fibrosis, SMC phenotypic alterations and inflammation response.
• SnRNA-seq reveals endothelial dynamics in human DCM and HCM.

## 1 Introduction

Cardiac dysfunction refers to a condition that the heart cannot pump effectively for the enough blood supply to the organs [1]. Studies have shown that lipids accumulation, inflammation response and cardiac fibrosis formation are the main features of cardiac dysfunction [1]. A high fat diet is rich in saturated and trans fats, a widely used methods to induce plaque buildup in coronary arteries and significantly increase the risk of heart diseases [2]. Cardiac dysfunction often accompanies with accumulation of apoptotic cells that is a main cause of secondary post-apoptotic necrosis and inflammatory response [2,3]. Efferocytosis is a process for the phagocytes to efficiently engulf apoptotic cells, mainly performed by professional phagocytes, such as macrophages, neutrophils, monocytes, and dendritic cells [4]. However, recent studies by us and other groups highlighted that aortic endothelial cells (ECs) have a high ability to perform efferocytosis via MerTK [[5], [6], [7], [8], [9], [10], [11], [12]]. Interestingly, as a main receptor for efferocytosis, MerTK has a similar expression level between macrophages and aortic ECs [[7], [8], [9], [10], [11]]. MerTK impairment leads to defective clearance of apoptotic cells and a propensity to autoimmunity. In the initiation of cardiac dysfunction, a variety of apoptotic cardiac cells (e.g., ECs, SMCs, cardiomyocytes, cardiac fibroblasts and circulating immune cells) are generated, stimulating the recruitment of macrophages and other immune cells and aggravating inflammation response [[1], [2], [3]]. Increasing evidence suggests that endothelial MerTK plays an important role in a variety of cardiovascular diseases (such as atherosclerosis, aortic aneurysm and vascular aging), lung disease and blood brain barrier disruption in neurological disorders [4]. However, the role of endothelial MerTK in cardiac dysfunction and its underlying mechanisms remain to be identified.

In this study, we performed multi-omics (including proteomics, scRNA-seq and snRNA-seq) and immunostaining in hearts from mouse models (*MerTK*^*flox/flox*^*Tie2*^*Cre*^ mice and WT mice) or scRNA-seq/snRNA-seq analysis from human specimens, to investigate the role of endothelial MerTK in cardiac dysfunction. This data provides compelling evidence that endothelial MerTK impairment represents a novel mechanism to induce cardiac dysfunction in the condition of high fat diet.

## 2 Methods

**Animals.** The *MerTK*^*flox/flox*^ mice and *Tie2-Cre* mice on the C57BL/6J background were generated by Cyagen US (Santa Clara, CA) and housed in the Division of Laboratory Animal Medicine at our institution with an approved ethic number of *A*23050. All experimental procedures were performed in accordance with protocols approved by the Institutional Animal Care and Use Committee and conformed to the Guidelines for the Care and Use of Laboratory Animals published by the US National Institutes of Health. The mice were maintained under a 12-h dark cycle at a controlled temperature of 21 ± 1 °C, with ad libitum access to water and a standard laboratory diet. *MerTK*^*flox/flox*^*Tie2*^*Cre*^ mice with MerTK conditional knockout in ECs were generated by crossing *MerTK*^*flox/flox*^ mice with *Tie2-Cre* mice (Cyagen US, Santa Clara, CA). The male *MerTK*^*flox/flox*^*Tie2*^*Cre*^ mice (n = 8) and their littermate control *MerTK*^*flox/flox*^ mice (n = 8) were injected with a single dose of AAV8-PCSK9 particles (1 × 10^11^) via the tail vein, followed by feeding with a high fat diet (HFD, TD.88137, Envigo) for two months, a widely used model simulating the environment of coronary atherosclerosis and subsequently accelerating cardiac dysfunction [12]. After mice were euthanized by CO_2_ asphyxiation, the heart tissues were carefully dissected from surrounding tissue, fixed with 10% neutral buffered formalin solution (Sigma, HT501128) and embedded in paraffin for immunohistochemical evaluation or stored in −80 °C for proteomics analysis.

**Proteomics measurements in the heart samples.** [[7], [8], [9], [10], [11]] Proteomics in the heart samples (n = 3 per group) were performed at the Georgia Tech Systems Mass Spectrometry Core. Following data acquisition, proteomic data were analyzed using an empirically corrected library and a quantitative analysis to obtain a comprehensive proteomic profile. Protein identification and quantification were performed using EncyclopeDIA, with results visualized with Scaffold DIA applying 1% false discovery thresholds at both the protein and peptide levels. Protein MS2 exclusive intensity values underwent quality assessment using ProteiNorm. The data were normalized by cyclic loss to perform statistical analysis using linear models for microarray data (limma) with empirical Bayes (eBayes) smoothing to the standard errors. Proteins with an FDR adjusted p-value <0.05 and a fold change >2 were considered significant. The proteomics data were analyzed by Qiagen IPA Software. All the IPA analyses for proteomics are available with detailed information in the Supplemental Data.

**Big data analytics and single-cell analysis in both mice and human patients. *Big data analytics*** for cardiac hypertrophy were based on the database from QIAGEN Ingenuity Pathway Analysis (IPA) and QIAGEN OmicSoft Land Explorer (OLE), which incorporate data from GEO (Gene Expression Omnibus), SRA (Sequence Read Archive), and ArrayExpress [[7], [8], [9], [10], [11]]. ***ScRNA-seq in mice*** for comparative function analysis of ECs and macrophages were based on the QIAGEN IPA database (1613- normal control [heart] NA CMP_ihc81kqbrwF5) that is associated with GEO DataSets of GSE176063 [13]. WT C57BL/6J mice, both male and female, were used for life stages ranging from the early embryonic stage to the mature adult stage [13]. A total about 520,000 single cells were collected and processed by scRNA-seq. Gene ontology (GO) biological pathway enrichment analysis for differentially expressed genes (DEGs) were performed [13]. The detailed methodology for big data analytics and scRNA-seq QIAGEN IPA and OLE were described in our recent publications [[7], [8], [9], [10], [11]]. ***SnRNA-seq in human*** comparative analysis for MerTK dynamics in ECs and macrophages from left ventricular tissue were based on the shared data from the Broad Institute's Single Cell Portal with the project ID SCP1303 and the raw sequence data from the dbGaP with the accession number of phs001539 [14]. R studio software was used for snRNA-seq analysis. In brief, 44 individuals, including 12 with DCM, 16 with HCM and 16 with non-failing (NF) hearts were collected from the patients. All patients with DCM and HCM exhibited advanced cardiomyopathy and required transplantation. A total of about 590,000 nuclei were used for snRNA-seq analysis. To avoid pseudo-replication bias inherent in single-cell data, all statistical comparisons were performed at the donor level using a pseudo-bulk approach. For each donor, the mean expression of each gene was calculated within the relevant cell type. Wilcoxon rank-sum tests were used for pairwise comparisons between disease groups (Control vs. DCM, Control vs. HCM, and DCM vs. HCM). Spearman correlation analysis was performed to assess the relationship between MerTK and key cardiac dysfunction-related genes at the donor level in endothelial cells. All analyses were performed in R (version 4.5.2) using ggplot2 for visualization. P < 0.05 was considered statistically significant.

**Cardiac fibrosis evaluation and immunofluorescent staining.** To evaluate cardiac fibrosis formation, Masson's Trichrome staining kit (Sigma) and Picrosirius Red staining kit (Abcam) were applied according to the provided protocols. Immunofluorescent staining was performed according to the protocol of ‘Immunofluorescent Staining of Paraffin-Embedded Tissue (Novus Biologicals)’ utilizing ReadyProbes™ Tissue Autofluorescence Quenching Kit (Invitrogen). The antibodies information is as follows: α-SMA, COLA1 and NF-kB were from Cell Signaling; Cav-1 was from Invitrogen; and ApoE was from Proteintech. Mean fluorescence intensity for protein expression was measured by ImageJ software.

**Statistical analysis.** An unpaired Student's t-test was used to determine statistical significance between two groups. Dunnett's one-way ANOVA was used for multiple comparisons between disease types and normal control. Data was analyzed with GraphPad Prism 9.4.1 and summarized as the mean ± SD. *P* < 0.05 was considered statistically significant.

## 3 Results

**Fig. 1:**
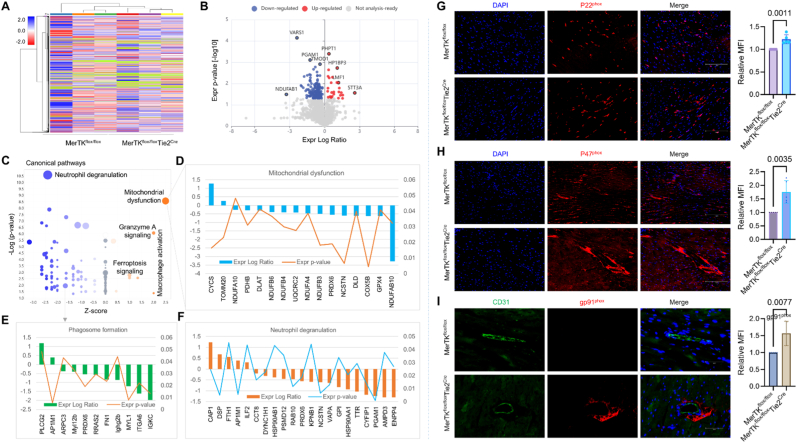
**Endothelial MerTK deficiency promotes cardiac dysfunction-related signaling pathways.** (**A**) Heat map for hierarchical clustering that was generated by Proteome Discoverer 3.2. (**B**) Volcano plot illustration for protein differentiation in the heart of *MerTK*^*flox/flox*^*Tie2*^*Cre*^ vs. *MerTK*^*flox/flox*^. Relative protein abundance (log2) plotted against significance level (-log10 P-value), showing downregulated (blue), upregulated (red) or non-differentially expressed proteins (grey). (**C–F**) The canonical pathways based on activation of z-score highlight activated mitochondrial dysfunction, and inhibited phagosome formation and neutrophil degranulation. Orange: positive value. Grey: no activity pattern. Size is based on the number of genes that overlap the pathway. (**G-I**) Immunostaining for the expression of NADPH oxidase and CD31 in the hearts from *MerTK*^*flox/flox*^ mice and *MerTK*^*flox/flox*^*Tie2*^*Cre*^ mice. *MerTK*^*flox/flox*^ mice and *MerTK*^*flox/flox*^*Tie2*^*Cre*^ mice were injected with a single dose of AAV8-PCSK9 particles along with a high fat diet for two months.

**Endothelial MerTK impairment promotes mitochondrial dysfunction.** Proteomics is a large-scale study of proteins (e.g., structure, function, and modifications), providing comprehensive understanding of changes in protein expression in heart diseases [15]. To investigate protein-protein interactions in endothelial MerTK-cardiac function, proteomics was employed in the hearts from *MerTK*^*flox/flox*^*Tie2*^*Cre*^ mice and *MerTK*^*flox/flox*^ mice. Fig. 1A shows the heat map in *MerTK*^*flox/flox*^*Tie2*^*Cre*^ mice and *MerTK*^*flox/flox*^ mice generated by Proteome Discoverer 3.2 (Thermo Fisher Scientific). The relative protein abundance indicates the representative proteins of each group that are clustered if they exhibit a similar expression trend across the samples. The hierarchical clustering was generated using neighbor joining algorithm with a Manhattan distance similarity measurement of the log2 ratios of the abundance of each sample relative to the average abundance. Fig. 1B is the volcano plot that illustrates 192 genes with a significant change (166 down and 26 up) analyzed by Qiagen IPA software. There is a clear separation in protein profiles between *MerTK*^*flox/flox*^*Tie2*^*Cre*^ mice and *MerTK*^*flox/flox*^ mice, showing highly changed proteins based on the Expr Log Ratio, including the upregulated proteins (e.g., PHPT1, HP1BP3, MF1 and STT3A) and the downregulated proteins (e.g. VARS1, PGAM1, TMOD1 and NDUFAB1). Our canonical pathways analysis highlights the key role of mitochondrial dysfunction, defective phagosome formation and increased neutrophil degranulation in endothelial MerTK deficiency-mediated cardiac dysfunction (Fig. 1C). Mitochondrial dysfunction can lead to cardiac dysfunction through mechanisms such as excessive production of reactive oxygen species (ROS), mitochondrial DNA (mtDNA) damage, and inflammasomes activation [16]. Our data shows that activated signaling of CYCS and TOMM20 as well as inhibited signaling of NDUFAB10 and GPX4 represent the main mechanisms in endothelial MerTK deficiency-mediated mitochondrial dysfunction (Fig. 1D). Phagosome formation is the key step in phagocytosis where a phagocyte's membrane extends to surround and engulfs a particle to create a vesicle [17]. Consistently our previous findings that endothelial MerTK inhibition promotes impaired efferocytosis, here we found that phagosome formation is inhibited in *MerTK*^*flox/flox*^*Tie2*^*Cre*^ group compared to *MerTK*^*flox/flox*^ group (Fig. 1E). Our data also showed that activated PLCG2 and AP1M1 as well as inhibited IGKC and ITGA6 are the main signaling involved in endothelial MerTK-mediated phagosome formation. This indicates that endothelial MerTK may have other functions to regulate phagocytosis, which needs to be further investigated. Neutrophil degranulation is the process by which neutrophils release the contents of their granules to fight bacterial infection or cause inflammation [18]. Neutrophil degranulation involves secretion of antimicrobial and inflammatory proteins, and excessive degranulation contributes to tissue damage [18]. Our data showed that, compared to *MerTK*^*flox/flox*^ group, *MerTK*^*flox/flox*^*Tie2*^*Cre*^ group demonstrates activated neutrophil granulation, indicating endothelial MerTK modulates inflammation response during the development of cardiac dysfunction (Fig. 1F). Furthermore, activated signaling (e.g., CAP1, DSP, and FTH1) and inhibited signaling (e.g., ENPP4, AMPD3 and PGAM1) play key roles in endothelial MerTK-mediated neutrophil degranulation. NADPH oxidases are specialized transmembrane enzymes that produce reactive oxygen species (ROS) by transferring electrons from NADPH to molecular oxygen [19]. Increased ROS from NADPH oxidases can trigger mitochondrial dysfunction, including reduced membrane potential, respiration and glutathione levels [19]. To validate the proteomics data on mitochondria dysfunction, we performed immunostaining for expression of NADPH oxidases (p22^phox^, p47^phox^, and gp^phox^) and CD31 (EC marker). As shown in Fig. 1G–I, compared to *MerTK*^*flox/flox*^ group, *MerTK*^*flox/flox*^*Tie2*^*Cre*^ group demonstrates enhanced expression of p22^phox^, p47^phox^, and gp^phox^ while decreased expression of CD31. These findings indicate that endothelial MerTK impairment is a key driver for oxidative stress generation and mitochondrial dysfunction, subsequently contributing to cardiac dysfunction.

**Fig. 2:**
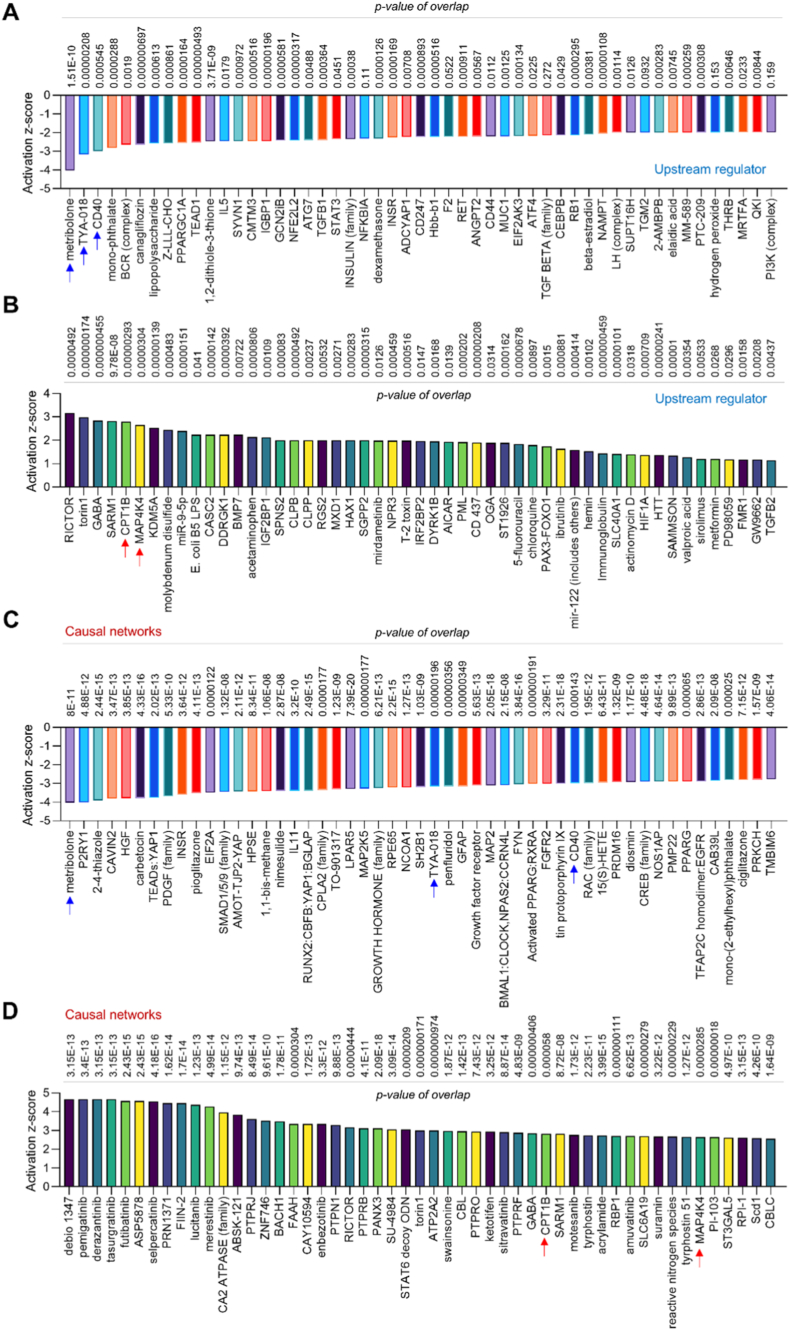
**Proteomics of upstream regulators and causal networks *MerTK*^*flox/flox*^*Tie2*^*Cre*^ vs. *MerTK*^*flox/flox*^.** (**A**-**B**) The top 50 activated and inhibited upstream regulators based on activation of z-score. (**C**-**D**) The top 50 upregulated and downregulated signaling in causal networks based on activation of z-score. *MerTK*^*flox/flox*^ mice and *MerTK*^*flox/flox*^*Tie2*^*Cre*^ mice were injected with a single dose of AAV8-PCSK9 particles along with a high fat diet for two months.

**IPA analysis for upstream regulators and causal networks.** IPA upstream regulator analysis is a reliable process to identify the upstream signaling that is potentially responsible for gene or protein changes observed in the experimental dataset. IPA causal network analysis is another process to expand upstream analysis that is not directly connected to targets in the dataset, further revealing the causal relationships associated with experimental data.Fig. 2A and B shows the downregulated and upregulated upstream regulators based on activation z-score in *MerTK*^*flox/flox*^*Tie2*^*Cre*^ group compared to *MerTK*^*flox/flox*^ group. Our upstream regulator analysis discovered inhibited signaling (e.g., CD40, PPARGC1A, TEAD1 and IL5) and activated signaling (e.g., RICTOR, MAP4K4, HIF1A and IGF2BP1) that may play an important role in endothelial MerTK-mediated cardiac dysfunction. Similarly, our causal network identified a variety of downregulated signaling pathways (e.g., P2RY1, EIF2A, MAP2K5, CD40) and upregulated signaling pathways (e.g., FIIN-2, RICTOR, PTPRJ and MAP4K4) that are involved in endothelial MerTK-mediated cardiac function (Fig. 2C and D). Interestingly, IPA analysis also shows the shared signaling between upstream regulators and causal networks, including the inhibited signaling pathways such as metribolone, TYA-018 and CD40 as well as activated signaling pathways such as CPT1B and MAP4K4. These shared signaling pathways indicate the potential mechanisms that may play key roles in endothelial MerTK-mediated cardiac function.

**Fig. 3:**
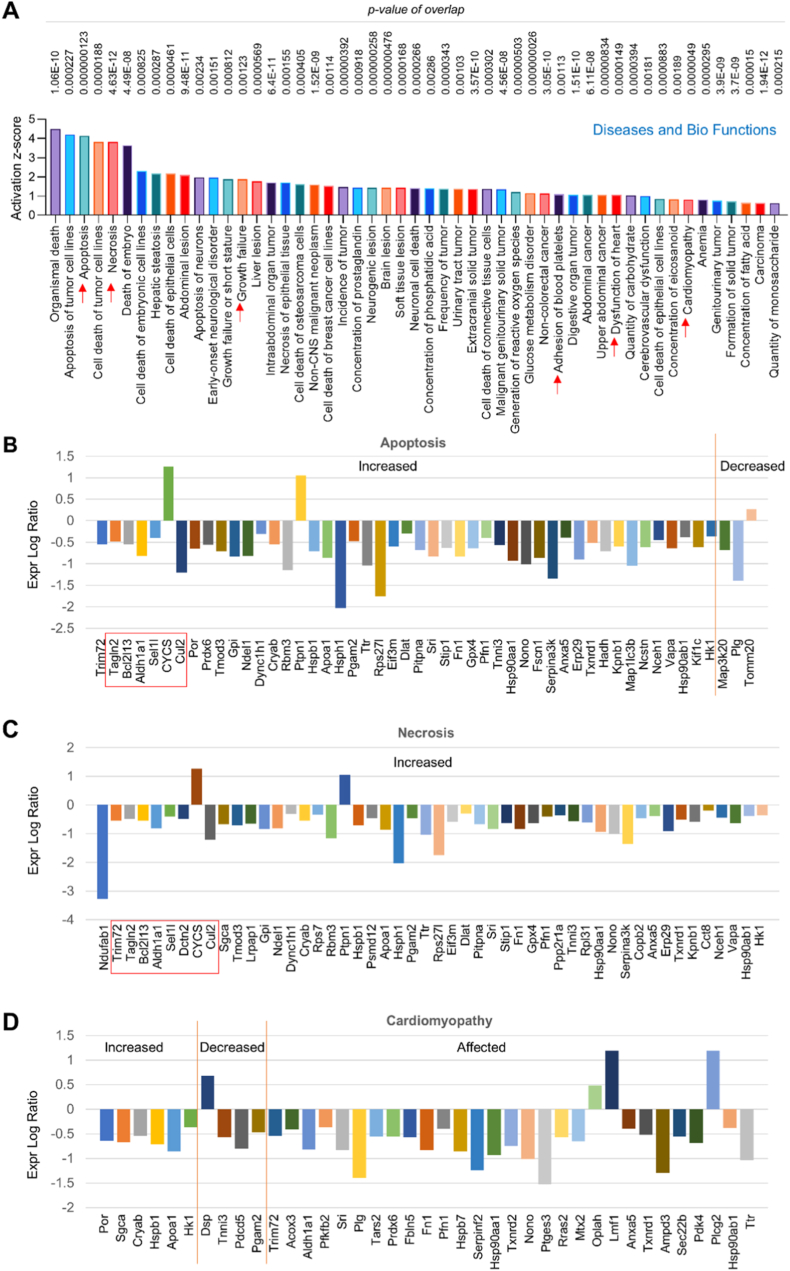
**Proteomics of Diseases and Bio Functions focus on activated signaling pathways.** (**A**-**D**) The top 50 upregulated signaling pathways based on activation of z-score, emphasizing activated apoptosis, necrosis and cardiomyopathy (*MerTK*^*flox/flox*^*Tie2*^*Cre*^ vs. *MerTK*^*flox/flox*^). *MerTK*^*flox/flox*^ mice and *MerTK*^*flox/flox*^*Tie2*^*Cre*^ mice were injected with a single dose of AAV8-PCSK9 particles along with a high fat diet for two months.

**IPA analysis for Diseases and Bio Functions in endothelial MerTK-mediated cardiac dysfunction.** This analysis is based on the literature compiled in the QIAGEN Knowledge Base using a z-score algorithm, predicting causal effects between molecules and downstream diseases and functions. Our analysis for upregulated Diseases and Bio Functions shows that endothelial MerTK deficiency significantly induces a variety of activated signaling pathways promoting cardiac dysfunction, including apoptosis, necrosis, cell growth failure, adhesion of blood platelet, and cardiomyopathy (Fig. 3A). Apoptosis causes the loss of cardiomyocytes that weakens the cardiac contractile function, contributing to ischemia/reperfusion injury, myocardial infarction, and subsequent heart failure [19]. MerTK is the main receptor of efferocytosis and MerTK inhibition has been shown to increase the accumulation of apoptotic cells [4]. To reveal apoptosis related signaling involved in endothelial MerTK-mediated cardiac dysfunction, Expr Log Ratio was used to quantify gene/protein expression. As shown in Fig. 3B, compared to *MerTK*^*flox/flox*^ group, *MerTK*^*flox/flox*^*Tie2*^*Cre*^ group demonstrates increased expression of apoptosis markers (e.g., Trim72, Ganln2 and Bcl2l13) and decreased expression of Map3k20, Pig and Tomm20. Different to apoptosis, necrosis is a form of cell injury caused by autolysis that leads to the cell death in tissues [20]. Both apoptosis and necrosis are two recognized cell death pathways that promote the death of cardiomyocytes [20]. For the necrosis, a variety of increased signaling was found in *MerTK*^*flox/flox*^*Tie2*^*Cre*^ group compared to *MerTK*^*flox/flox*^ group, including Ndufab1, Trim72, Tagln2 and Bcl2l13 (Fig. 3C). Interestingly, a series of shared signaling between apoptosis and necrosis were found, including Tagln2, Bcl2l13, Bcl2l13, Aldh1a1, Sel11, CYCS and Cul2. Cardiomyopathy is conventionally defined as systolic dysfunction caused by coronary artery disease or abnormal loading conditions. Our data showed that activated signaling (e.g., Por, Sqca and Cryab) and inhibited signaling (e.g., Dsp, Tnni3 and Pdcd5) are involved in endothelial MerTK-mediated cardiac function (Fig. 3D). Meanwhile, a variety of signaling was found that is affected by endothelial MerTK deficiency, including Adh1a1, Plg, Lmf1 and Pcg2.

**Fig. 4:**
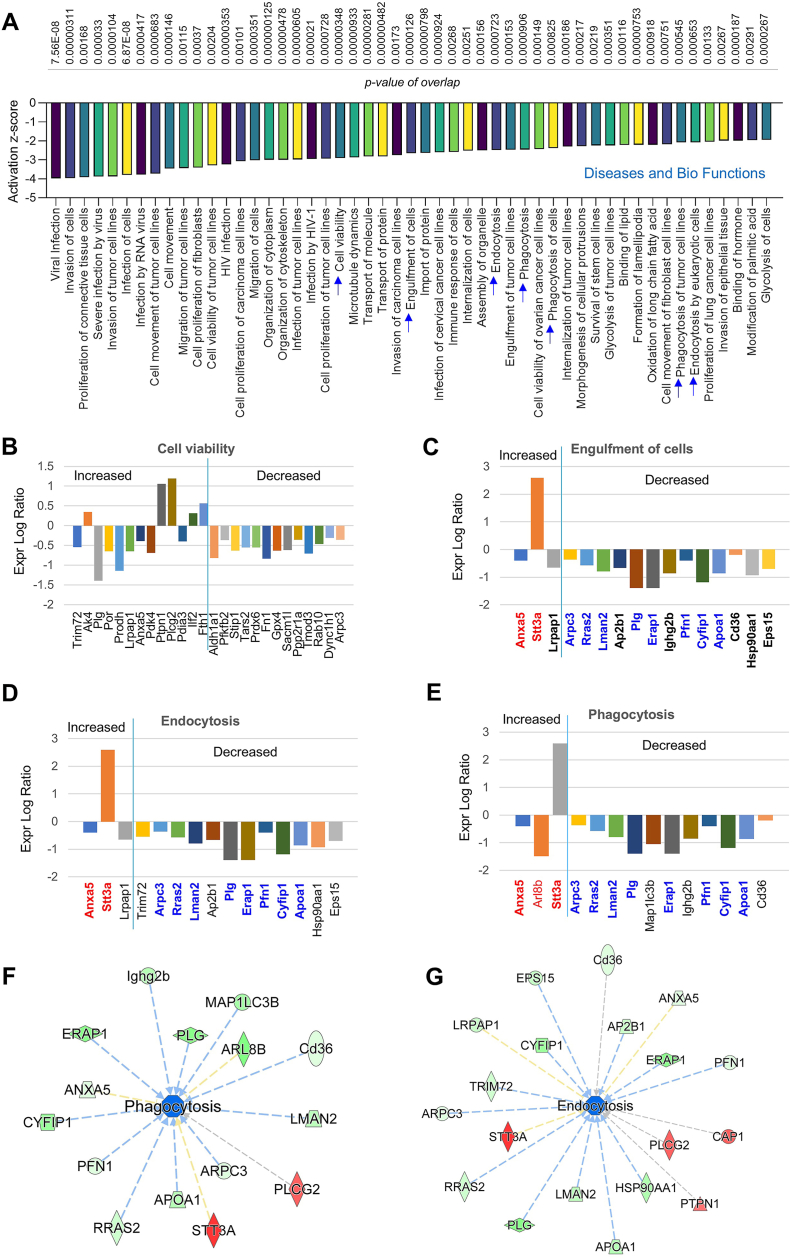
**Proteomics of Diseases and Bio Functions analysis for inhibited signaling pathways.** (**A**-**E**) The top 50 inhibited signaling pathways based on activation of z-score (*MerTK*^*flox/flox*^*Tie2*^*Cre*^ vs. *MerTK*^*flox/flox*^), highlighting the decreased activities of cell viability, engulfment of cells, endocytosis, phagocytosis. (**F**-**G**) IPA prediction for the pathways of phagocytosis and endocytosis. Upregulated and downregulated proteins are highlighted in red and green, respectively, and the color depth is correlated to the fold change. *MerTK*^*flox/flox*^ mice and *MerTK*^*flox/flox*^*Tie2*^*Cre*^ mice were injected with a single dose of AAV8-PCSK9 particles along with a high fat diet for two months.

Consistently, our analysis for downregulated Diseases and Bio Functions showed that efferocytosis related signaling (e.g., cell viability, engulfment of cells, endocytosis and phagocytosis) is markedly inhibited in *MerTK*^*flox/flox*^*Tie2*^*Cre*^ group compared to *MerTK*^*flox/flox*^ group (Fig. 4A). This indicates that MerTK plays a key role in regulating efferocytosis in ECs. For the cell viability, we found that endothelial MerTK deficiency induces expression of Trim72, Ak4 and Por and inhibits expression of Adh1a1, Pfkfb2 and Stip1 (Fig. 4B). More interestingly, for the deep analysis of engulfment of cells, endocytosis and phagocytosis, a series of shared signaling was found in *MerTK*^*flox/flox*^*Tie2*^*Cre*^ group compared to *MerTK*^*flox/flox*^ group, including activated signaling (e.g., Anxa5 and Stt3a) and inhibited signaling (e.g., Arpc3, Rras2, Lman2, Plg, Erap1, Pfn1, Cyfp1 and Apoa1) (Fig. 4C–E). In consistence, the IPA analysis also predicted the networks of phagocytosis and endocytosis, including activated signaling of STT3A and PLCG2 as well as inhibited signaling of ERAP1 and PLG (Fig. 4F and G).

**Fig. 5:**
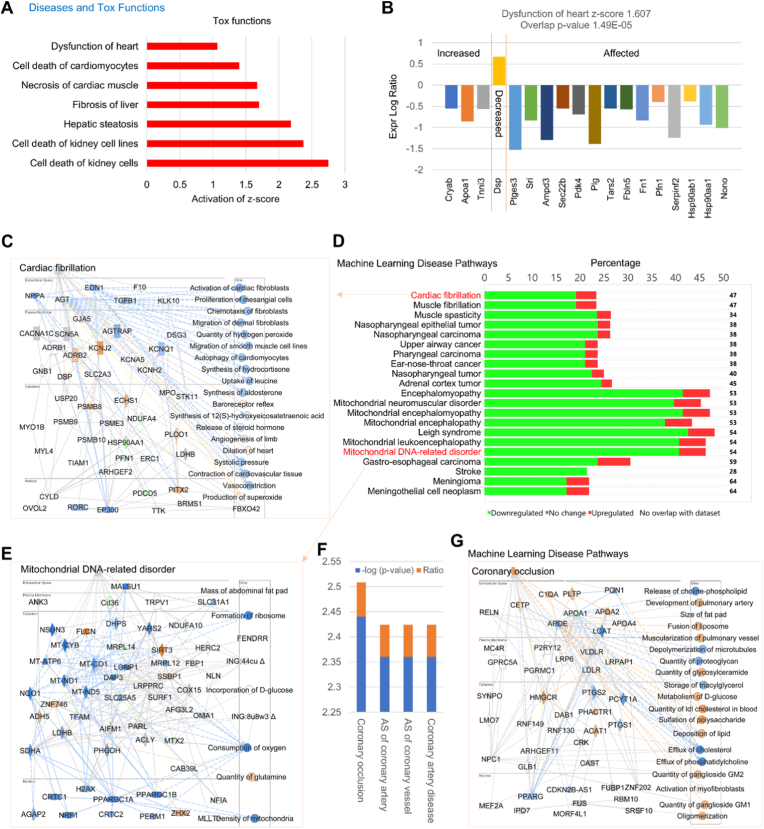
**Proteomics of Tox functions and Machine Learning Disease Pathways *MerTK*^*flox/flox*^*Tie2*^*Cre*^ vs. *MerTK*^*flox/flox*^.** (**A**) IPA for Diseases and Tox functions analysis based on activation of z-score. (**B**) The signaling that is involved in dysfunction of heart. (**C**-**E**) Overall Machine Learning Disease pathways with detailed information about cardiac fibrillation and mitochondrial DNA-related disorder. (**F**-**G**) Machine Learning Disease pathways in cardiac dysfunction focusing on coronary occlusion and atherosclerosis (AS) of coronary arteries. *MerTK*^*flox/flox*^ mice and *MerTK*^*flox/flox*^*Tie2*^*Cre*^ mice were injected with a single dose of AAV8-PCSK9 particles along with a high fat diet for two months.

**IPA analysis for Diseases and Tox Functions and Machine Learning Disease Pathways.** As described at the website of Qiagen IPA, toxicity functions relate to hepatotoxicity, nephrotoxicity, or cardiovascular toxicity and mark clinical pathology endpoints. IPA can score the dataset and rank these functions as well as running a full functional analysis. Here, we mainly focused on the role of endothelial MerTK deficiency in cardiac functions based on activation of z-score. As shown in Fig. 5A, our data showed that endothelial MerTK deficiency promotes dysfunction of heart, cell death of cardiomyocytes and necrosis of cardiac muscle. Meanwhile, we also found that endothelial MerTK deficiency activates a variety of liver disorders, including fibrosis of liver, hepatic steatosis, cell death of kidney cells. With the deep analysis for dysfunction of heart, we found that *MerTK*^*flox/flox*^*Tie2*^*Cre*^ group demonstrates increased expression of Cryab, Apoa1 and Tnni3, and decreased expression of Dsp compared to *MerTK*^*flox/flox*^ group (Fig. 5B).

To further clarify the role of endothelial MerTK in regulating cardiac function, we performed Machine Learning Disease Pathways analysis based on QIAGEN IPA software and IPA database. Our analysis indicates that, compared to *MerTK*^*flox/flox*^ group, *MerTK*^*flox/flox*^*Tie2*^*Cre*^ group shows activated cardiac fibrillation and mitochondrial DNA-related disorders, both play important roles in cardiac dysfunction (Fig. 5C). For the cardiac fibrillation, our deep analysis shows activated signaling (e.g., KCNJ2, ADRB2, DSP, PLOD1 and PITX2) and inhibited signaling (e.g., NPPA, EDN1, TGFβ1 and RORC) in *MerTK*^*flox/flox*^*Tie2*^*Cre*^ group compared to *MerTK*^*flox/flox*^ group. Meanwhile, a series of mechanism pathways were found in *MerTK*^*flox/flox*^*Tie2*^*Cre*^ group compared to *MerTK*^*flox/flox*^ group, including inhibited autophagy of cardiomyocytes and contraction of cardiac tissue as well as activated dilation of heart and production of superoxide, all are key drivers to promote cardiac dysfunction (Fig. 5D) [[1], [2], [3]]. For mitochondrial DNA-related disorders, we found that activated signaling (e.g., FLCN, SIRT3, ADH5 and ZNF746), inhibited signaling (e.g., NOD1, NSON3- which codes for eNOS, and PPARGC1A), and inhibited pathways (e.g., formation of ribosome, consumption of oxygen and density of mitochondria, Fig. 5E). Interestingly, our further analysis focusing on cardiovascular biology shows that *MerTK*^*flox/flox*^*Tie2*^*Cre*^ group demonstrates activated coronary occlusion, atherosclerosis (AS) of coronary artery and vessel, and coronary artery disease (Fig. 5F). For coronary occlusion, our deep analysis indicates that endothelial MerTK deficiency shows activated signaling (e.g., C1QA, PLTP and HMGCR) and inhibited signaling (e.g., APOE, LDLR and LCAT) as well as upregulated pathways (e.g., quantity of LDL cholesterol in blood, fusion of liposome and deposition of lipid) and downregulated pathways (e.g., efflux of cholesterol, quantify of glucosylceramide and release of choline-phospholipid, Fig. 5G).

**Fig. 6:**
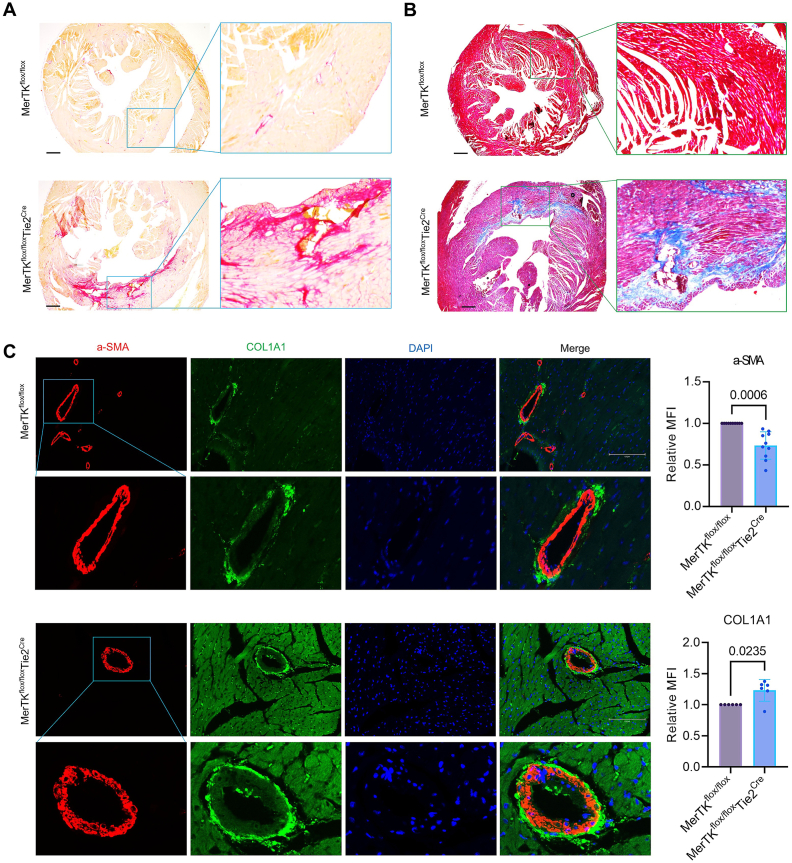
**Cardiac fibrosis evaluation and immunostaining validation for cardiac dysfunction related signaling.** (**A**-**B**) Picrosirius Red staining and Masson's Trichrome staining in the hearts from *MerTK*^*flox/flox*^ mice and *MerTK*^*flox/flox*^*Tie2*^*Cre*^ mice. (**C**) Immunostaining for the expression of α-SMA and COL1A1 in the hearts from *MerTK*^*flox/flox*^ mice and *MerTK*^*flox/flox*^*Tie2*^*Cre*^ mice. *MerTK*^*flox/flox*^ mice and *MerTK*^*flox/flox*^*Tie2*^*Cre*^ mice were injected with a single dose of AAV8-PCSK9 particles along with a high fat diet for two months.

**Endothelial MerTK deficiency promotes cardiac dysfunction.** Our proteomics highlight a novel concept that endothelial MerTK impairment regulates a variety of signaling pathways, promoting cardiac dysfunctions. To validate our proteomics findings, we performed immunostaining in the hearts from *MerTK*^*flox/flox*^ mice and *MerTK*^*flox/flox*^*Tie2*^*Cre*^ mice. Cardiac fibrosis is a process for the excessive buildup of scar tissue such as collagens in the heart that can directly cause cardiac dysfunction by stiffening the muscle and impairing its ability to contract and relax properly [21]. Picrosirius Red staining and Masson's Trichrome staining are two commonly used methods to detect visualizing collagen deposition in cardiac tissues, an indicator for cardiac fibrosis formation [22]. Our data with both Masson's Trichrome staining and Picrosirius Red staining showed that, compared to *MerTK*^*flox/flox*^ group, *MerTK*^*flox/flox*^*Tie2*^*Cre*^ group demonstrates extremely higher collagen deposition in the heart (Fig. 6A and B). In addition, *MerTK*^*flox/flox*^*Tie2*^*Cre*^ group demonstrates much higher cardiac hypertrophy compared to *MerTK*^*flox/flox*^*Tie2*^*Cre*^ group. Consistently, our immunostaining for COL1A1 (Collagen Type I Alpha 1, a known marker for collogen deposition) showed that COL1A1 expression is much higher in *MerTK*^*flox/flox*^*Tie2*^*Cre*^ group than that in *MerTK*^*flox/flox*^ group (Fig. 6C). Smooth muscle cell (SMC) phenotypic switching refers to SMCs shift from a contractile state to a proliferative and synthetic state, representing another marker for cardiac dysfunction [23]. Our data showed that SMC contractile marker of α-SMA expression decreases while synthetic marker of CLO1A1 increases in *MerTK*^*flox/flox*^*Tie2*^*Cre*^ group compared to *MerTK*^*flox/flox*^ group (Fig. 6C). These findings indicate that endothelial MerTK deficiency promotes cardiac fibrosis formation, cardiac hypertrophy, and SMC phenotypic switching, contributing to cardiac dysfunction.

**Fig. 7:**
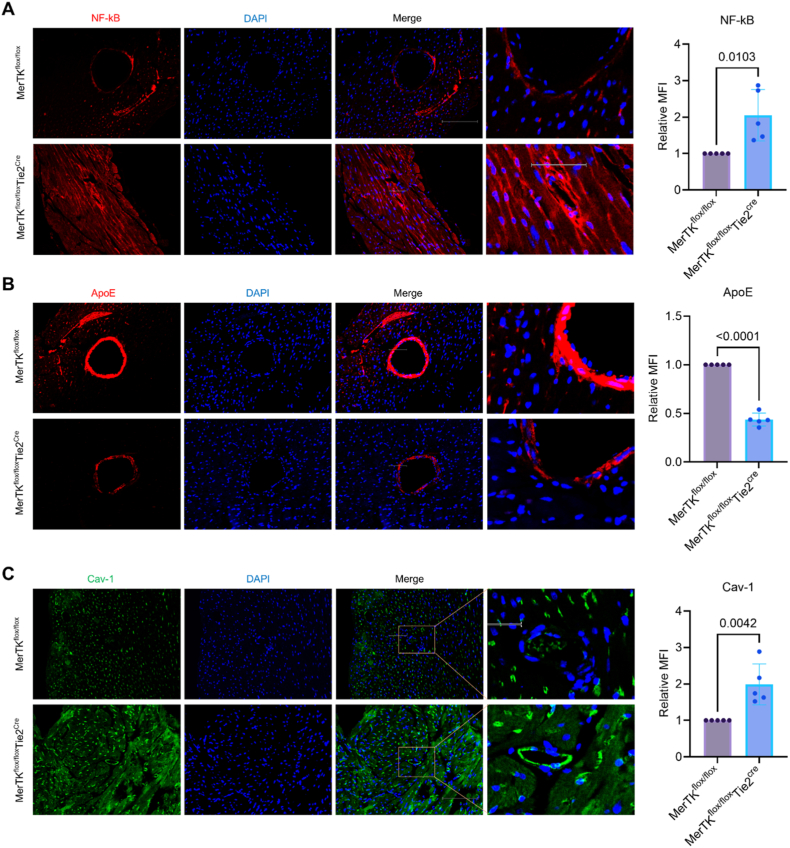
**Endothelial MerTK deficiency promotes signaling pathways contributing to cardiac dysfunction.** (**A**-**C**) Immunostaining for the expression of NF-kB, ApoE and Cav-1 in the hearts from *MerTK*^*flox/flox*^ mice and *MerTK*^*flox/flox*^*Tie2*^*Cre*^ mice. *MerTK*^*flox/flox*^ mice and *MerTK*^*flox/flox*^*Tie2*^*Cre*^ mice were injected with a single dose of AAV8-PCSK9 particles along with a high fat diet for two months.

Besides α-SMA and CLO1A1, NF-kB, ApoE (apolipoprotein E) and Cav-1 (caveolin 1) are other widely used indicators for cardiac function [24]. NF-kB is a crucial transcription factor that drives chronic inflammation by activating pro-inflammatory genes such as IL-6 and TNF-α [25]. ApoE helps clear cholesterol-rich lipoproteins in blood that is a crucial step for preventing plaque buildup [26]. Cav-1 regulates cholesterol, inflammation, and nitric oxide (NO) pathways, with loss of Cav-1 leading to activated expression endothelial nitric oxide synthase (eNOS) and increased production of NO as well as inhibited inflammation and reduced low-density lipoprotein trafficking [27], [28]; all are main factors contributing to cardiac function [[1], [2], [3]]. Our immunostaining data showed that, compared to *MerTK*^*flox/flox*^ group, *MerTK*^*flox/flox*^*Tie2*^*Cre*^ group demonstrates much higher expression of NF-kB and Cav-1 while lower expression of ApoE (Fig. 7A–C). These findings further confirm that endothelial MerTK impairment is an important driver to promote cardiac dysfunction in the condition of high fat diet.

**Fig. 8:**
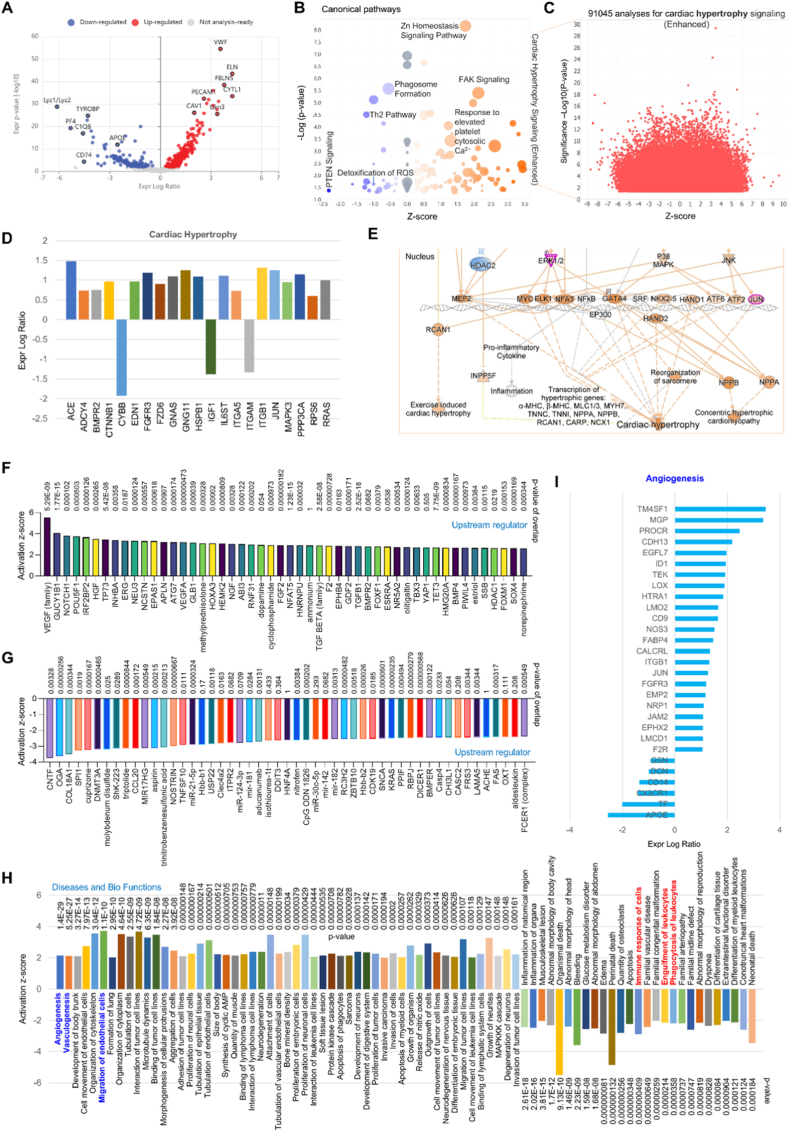
**ScRNA-seq analysis and big data analytics in cardiac hypertrophy.** (**A**) Volcano plot illustration for gene differentiation in the hearts from seven age groups of WT C57BL/6J mice. (**B**) Canonical pathways based on activation of z-score and -Log (p-value). (**C**) Big data analytics for 91045 analyses of cardiac hypertrophy signaling based on QIAGEN IPA database. (**D-E**) The key signaling pathways involved cardiac hypertrophy. (**F-G**) Upstream regulator analysis for activated or inhibited signaling molecules (ECs vs. macrophages). (**H**) QIAGEN IPA for Diseases and Bio Functions analysis based on activation of z-score. (**I**) The key signaling molecules involved in angiogenesis.

**ScRNA-seq analysis for the comparison between ECs and macrophages and big data analytics for cardiac hypertrophy.** ECs and macrophages are both phagocytes that can clear pathogens, cell debris, and apoptotic cells; however, macrophages are “professional” phagocytes while ECs act as “non-professional” gatekeepers [4]. To clarify the difference for the functions between ECs and macrophages, we performed the Match Analysis in QIAGEN IPA database based on our proteomics data. We identified a matched scRNA-seq project with 10 heart tissues with seven age groups of WT C57BL/6J mice, ranged from embryonic day (E) 10.5, E12.5, E14.5, postnatal day (P) 0, P10, P21 to adult stage [13]. This wide range of age stages provides a perfect platform to analyze the function differences between ECs and macrophages in the viewpoint of developmental biology. First, our data for the volcano plot illustration indicated that there are 500 genes passed cutoffs (Expr Other >1.0) with a clear separation in gene profiles (149 down and 351 up), showing highly changed proteins based on the Expr Log Ratio, including the upregulated proteins (e.g., VWF, ELN, PECAM, and CAV1) and the downregulated proteins (e.g. Lyz1/Lyz2, TYROBP, APOE and PF4, Fig. 8A). Second, the canonical pathways highlighted the activated signaling pathways (e.g., Zn homeostasis, PAK and response to elevated platelet cytosolic Ca^2+^) and inhibited pathways (e.g., phagosome formation, Th2 pathway, and detoxification of ROS) when compared ECs to macrophages (Fig. 8B). Interestingly, we found that enhanced cardiac hypertrophy signaling represents a main mechanism in ECs compared to macrophages. We thereby performed unique big data analytics based on QIAGEN IPA database with 91045 cross-analysis for cardiac hypertrophy in mammals (rats, mice and humans, Fig. 8C). Our big data analytics revealed a series of genes with significant changes in ECs vs. macrophages, such as ACE, ADCY4, BMPR2 and CYBB based on Expr Log Ratio (Fig. 8D). More interestingly, we found that activation of MAPK family, including ERK12, P38 MAPK and JNK, represents a main mechanism in cardiac hypertrophy (Fig. 8E). The upstream regulator analysis revealed that activated signaling (e.g., VEGF family, NOTCHI1, and HGF) and inhibited signaling (e.g., CNTF, CGA and COL18A1) may determine the function differences between ECs and macrophages in cardiac hypertrophy (Fig. 8F and G). The Diseases and Bio Functions analysis showed that activated endothelial functions (e.g., angiogenesis, vasculogenesis, and EC movement), and inhibited macrophage-mediated functions (e.g., phagocytosis, inflammation and immune response) are predominant pathways in cardiac hypertrophy (Fig. 8H). Finally, we performed deep analysis for the signaling involved in angiogenesis, including activated signaling of TM4SF1, MGP and PROCR, and inhibited signaling of APOE, TF and CX3CR1 (Fig. 8I). These findings provide a novel viewpoint for the function differences between ECs and macrophages in cardiac hypertrophy based on single-cell analysis.

**Fig. 9:**
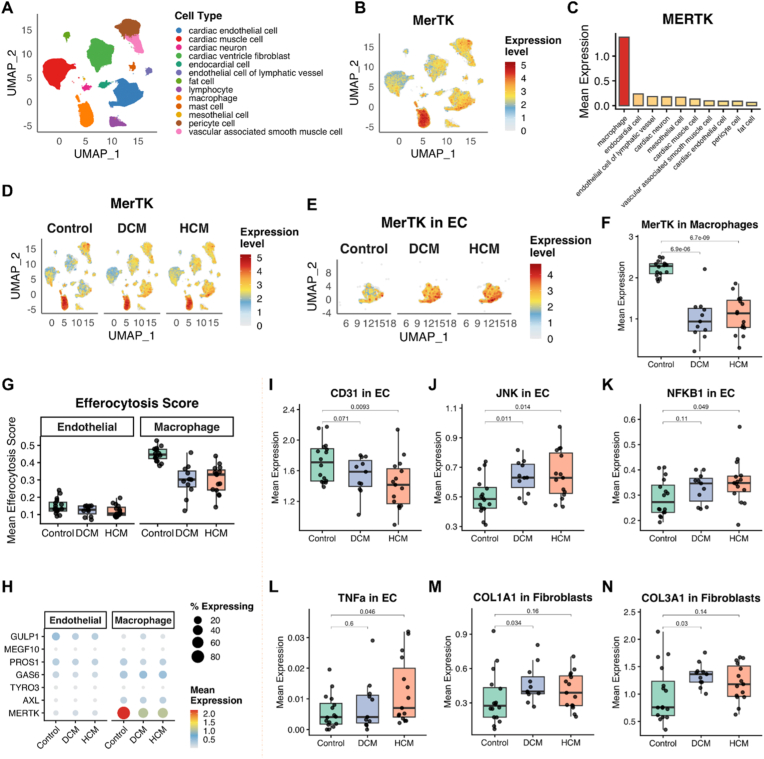
**SnRNA-seq analysis for MerTK expression and efferocytosis-related signaling in human dilated and hypertrophic cardiomyopathy.** (**A**) Uniform Manifold Approximation and Projection (UMAP) visualization of 592,689 nuclei from 42 human left ventricles (16 Control, 11 DCM, 15 HCM), colored by cell type. Thirteen distinct cell populations were identified. (**B**) UMAP visualization of MerTK gene expression across all nuclei. (**C**) Mean MerTK expression across the top 10 cell types. (**D**) MerTK expression on UMAP split by disease condition. (**E**) MerTK expression in endothelial cells (cardiac endothelial cells, endocardial cells, and endothelial cells of lymphatic vessels) split by disease condition. (**F**) Pseudo-bulk analysis of MerTK expression in macrophages. Each dot represents one donor. (**G**) Pseudo-bulk analysis for comparison of efferocytosis signature scores between endothelial cells and macrophages. The efferocytosis score was calculated as the mean expression of seven efferocytosis-related genes (MERTK, AXL, TYRO3, GAS6, PROS1, MEGF10, GULP1). (**H**) Dot plot of efferocytosis-related genes comparing endothelial cells and macrophages across disease groups. Dot size represents the percentage of expressing cells; color intensity represents mean expression level. (**I**-**L**) Pseudo-bulk analysis for expression of CD31, JNK, NFKB1 and TNFα in ECs. (**M**-**N**) Pseudo-bulk analysis for expression of COL1A1 and COL3A1 in cardiac ventricle fibroblasts.

**SnRNA-seq reveals the mechanisms of endothelial MerTK in human cardiac hypertrophy.** To explore the underlying mechanisms for endothelial MerTK in cardiac hypertrophy and make our research more clinically relevant, we reanalyzed a publicly available snRNA-seq dataset [14]. This dataset comprises 592,689 nuclei isolated from left ventricular tissue of 42 individuals, including 16 non-failing controls (Control), 11 patients with dilated cardiomyopathy (DCM), and 15 patients with hypertrophic cardiomyopathy (HCM). The processed log-normalized expression matrix, cell barcodes, gene annotations, cell-type metadata, and UMAP coordinates were downloaded from the Broad Institute Single Cell Portal (SCP1303). Expressions of MerTK and other interesting genes were extracted from the sparse expression matrix. Cell-type annotations and disease group assignments were obtained from the provided metadata. ECs were defined as the combined population of cardiac endothelial cells, endocardial cells, and endothelial cells of lymphatic vessels. Among the 592,689 qualified nuclei in left ventricular from 42 individuals, 13 clusters were identified including cardiac ECs, cardiomyocytes (cardiac muscle cell), fibroblasts and macrophages (Fig. 9A). Next, we evaluated MerTK expression in these 13 clusters, indicating that MerTK is highly expressed in ECs and macrophages (Fig. 9B and C). It's worth noting that the overall expression of MerTK is decreased in both DCM and HCM compared to control group (Fig. 9D). However, MerTK has a completely different expression pattern in ECs compared to macrophages. As shown in Fig. 9E and F, MerTK expression is increased in ECs while decreased in macrophages from both DCM and HCM groups compared to control group. Our efferocytosis score analysis showed that efferocytosis in both ECs and macrophages is impaired in DCM and HCM compared to control group (Fig. 9G). Then we performed deep analysis of efferocytosis-related genes in ECs and macrophages, including TAM receptor family (TYRO3, AXL and MERTK), TAM receptor ligation (GAS6 and PROS1), MEGF10 (multiple EGF-like domains 10, a crucial class F scavenger receptor that mediates efferocytosis), and GULP1 (phagocytic adaptor protein). In ECs, expressions of GULP1, PROS1 and GAS6 is markedly decreased; while in macrophages, only MerTK expression is significantly inhibited (DCM/HCM vs. control, Fig. 9H). These findings indicate that MerTK may have other functions in addition to efferocytosis and the functional differences between non-professional phagocytes of ECs and professional phagocytes of macrophages.

Pseudo-bulk analysis in snRNA-seq aggregates single-cell data to create a “bulk-like” profile by summing gene counts within specific clusters and biological replicates, reducing technical noise and mitigating inflated p-values. To clarify the role of ECs in cardiac hypertrophy, we focused on a series of genes that are highly expressed in ECs, including CD31, JNK, NFKB1 and TNFα. CD31, also known as platelet endothelial cell adhesion molecule (PECAM-1), is critical marker for vascular ECs [29]. JNK in ECs acts as a critical, flow-sensitive, and matrix-dependent mediator of inflammatory responses and vascular homeostasis [30]. NFKB1 and TNFα are the primary mediators of the endothelial inflammatory response, translating external stimuli into the expression of adhesion receptors and promoting monocytes recruitment [31]. Our data showed that, compared to control group, expression of CD31 decreases while expression of JNK, NFKB1 and TNFα in both DCM and HCM groups (Fig. 9I–L). Collagen deposition is a critical component of cardiac hypertrophy [32]. COL1A1 and COL3A1 are the main types of collagens that are highly expressed in cardiac fibroblasts [32]. As expected, our data showed that expression of both COL1A1 and COL3A1 increases in DCM and HCM groups compared to control groups (Fig. 9M − N). Overall, our snRNA-seq analysis clarify that impaired endothelial efferocytosis along with decreased expression of EC marker CD31 and increased expression of cardiac dysfunction markers, including JNK, NFKB1, TNFα, COL1A1 and COL3A1.

## 4 Discussion

A high fat diet is linked to obesity and leads to the buildup of plaque in coronary arteries, a primary cause of cardiac dysfunction and subsequent heart failure [[1], [2], [3]]. A main feature of cardiac dysfunction is the accumulation of apoptotic cells in the intimal space of EC monolayer, resulting in the secondary necrotic cell debris formation [[1], [2], [3]]. It has been shown that necrotic cell debris is a powerful trigger for pro-inflammation through releasing intracellular components of damage-associated molecular patterns, including DNA, histones, and ATP that alert the immune system [33]. Increasing evidence validates that aortic ECs have a high capacity to perform efferocytosis for the efficient clearance of apoptotic cells [[5], [6], [7], [8], [9], [10], [11]]. As a TAM family, MerTK is the key receptor for efferocytosis and determines the efferocytosis ability in phagocytes including macrophages and aortic ECs [4]. MerTK expression is inhibited in the condition of high fat diet, mainly because of increased concentrations of oxidized low-density lipoprotein (ox-LDL) and secreted pro-inflammatory cytokines [3]. Endothelial MerTK plays an important role in a variety of cardiovascular diseases (e.g., atherosclerosis, aortic aneurysm and vascular aging), blood brain barrier-mediated brain disorders and lung diseases [[5], [6], [7], [8], [9], [10], [11]]. However, it remains unknown for the endothelial role MerTK in cardiac function particularly in the condition of high fat diet. In this study, we integrated multi-omics (proteomics, scRNA-seq and snRNA-seq) with immunostaining validation in the heart tissues from *MerTK*^*flox/flox*^ mice and *MerTK*^*flox/flox*^*Tie2*^*Cre*^ mice. Our findings revealed that endothelial MerTK impairment markedly induces cardiac fibrosis formation and cardiac hypertrophy, accelerating the progression of cardiac dysfunction. We also identified a series of signaling pathways in endothelial MerTK deficiency-mediated cardiac dysfunction, including activated mitochondrial dysfunction, apoptosis and necrosis and inhibited cell viability, phagosome formation, and phagocytosis. Our immunostaining further indicates that cardiac fibrosis formation, SMCs phenotypic switching, lipids deposition and inflammation are key mechanisms of endothelial MerTK in cardiac dysfunction.

Cardiac fibrosis refers to a process of excessive deposition of extracellular matrix (ECM) proteins by cardiac fibroblasts that accelerate the development of heart failure [[1], [2], [3]]. Among numerous of inflammatory cytokines, TGFβ1 is a pivotal driver of cardiac fibrosis [[1], [2], [3]]. Meanwhile, TGFβ1 is also a crucial anti-inflammatory cytokine induced by efferocytosis to promote inflammation resolution, cardiac repair and tissue homeostasis^.34^ This difference between TGFβ1 in activation of cardiac fibrosis and efferocytosis in secretion of TGFβ1 raise an interesting question about the genuine role of efferocytosis in cardiac fibrosis [4,34]. To answer this question, we summarized the key mechanisms of TGFβ1-MerTK-efferocytosis axis in macrophages and its contribution to cardiac fibrosis [4,35,36]. First, TGFβ1 activates the differentiation of cardiac fibroblasts into myofibroblasts that deposit extracellular matrix and promote cardiac fibrosis. Second, macrophage MerTK induces TGFβ1 production and creates a positive feedback loop that drives tissue fibrosis, particularly when MerTK is cleaved or its function is impaired, leading to poor inflammation resolution and worse tissue fibrosis. Consistent with the role of macrophage MerTK in cardiac fibrosis, our data indicate that endothelial MerTK deficiency significantly promotes cardiac fibrosis formation. However, in addition to TGF-β1, mechanisms such as SMC phenotypic alterations, collagen deposition and inflammation may represent other MerTK functions in the development of cardiac fibrosis [37]. Combined with our previous findings that EC MerTK promotes endothelial dysfunction in atherosclerosis, aortic aneurysm and vascular aging [[7], [8], [9], [10], [11]]; it can be concluded that endothelial MerTK may represent a novel therapeutic target to prevent these cardiovascular diseases.

Professional phagocytes such as macrophages and neutrophils are immune cells specialized for highly efficient and rapid engulfment of pathogens/debris [4,38]. Compared with professional phagocytes, non-professional phagocytes (e.g., aortic ECs and epithelial cells) perform phagocytosis inefficiently as a secondary function for tissue repair [4,38]. Non-professional phagocytes often have a slower phagocytosis that is triggered by specific late-stage “eat-me” signals from dying cells, ensuring timely clearance when professional phagocytes are scarce [4,38]. Aortic ECs are traditional non-professional phagocytes, however, increasing evidence has validated that aortic ECs have a high ability to perform efferocytosis, similar to that in macrophages [[5], [6], [7], [8], [9], [10], [11]]. Aortic ECs act macrophage-like gatekeepers to clear dying cells (e.g., red blood cells, apoptotic immune cells, and neighboring SMC debris) and pathogens [[5], [6], [7], [8], [9], [10], [11]]. Efficient efferocytosis in aortic ECs is crucial for vascular homeostasis and prevents chronic inflammation, which are main features in a variety of cardiovascular diseases including atherosclerosis, vascular aging and myocardial infarction [[5], [6], [7], [8], [9], [10], [11]]. Interestingly, compared to macrophages, efferocytosis in aortic ECs varies based on their location and is regulated by blood flow patterns [[4], [5], [6], [7], [8], [9], [10], [11],^38^]. Defective efferocytosis in both professional phagocytes and non-professional phagocytes is linked to human diseases, including cardiovascular diseases, cancer and brain disorders [4,38]. Most interestingly, the importance of endothelial MerTK has been recognized by more research groups highlighting its critical role in cardiovascular diseases (e.g., atherosclerosis, aortic aneurysm and vascular aging), blood–brain barrier in neuronal disorders and lung disease [[4], [5], [6], [7], [8], [9], [10], [11],^38^]. The main mechanisms of endothelial efferocytosis involve in endothelial dysfunction, increased SMC phenotypic alterations from a contractile state to a variety of alternative functional states, and abnormal redox activities [[4], [5], [6], [7], [8], [9], [10], [11],^38^]. All these mechanisms are closely associated with cardiac dysfunction.

It is worth noting that MerTK may have other functions in addition to efferocytosis in cardiac ECs based on our snRNA-seq analysis in human hearts. The most extensively studied function of MerTK is its regulation of efferocytosis, which is crucial for maintaining tissue health and inflammation resolution [4,38]. The initiation of efferocytosis originates from apoptotic cells releasing “find me” signals to phagocytes, such as CX3CL1, S1P lipids, lysophosphatidylcholine, and ATP and UTP nucleotides [4,38]. These “Find Me” signals ensure the rapid clearance of appototic cells. Apoptotic cells to be cleared must express “Eat Me” signals such as phosphatidylserine (PtdSer) that prompt phagocytes to engulf them [4,38]. On the other hand, healthy cells express “Don't Eat Me” signals such as CD47 and CD31 that prevent phagocyte activation and phagocytosis [4,38]. To facilitate the binding of apoptotic cells to phagocytes, bridging molecules (such as GAS6 and protein S) bind to phosphatidylserine (PtdSer) exposed on the surface of apoptotic cells and activate MerTK receptors (or TYRO3 and AXL) on phagocytes [4,38]. Activated MerTK allows phagocytes to extend their cell membranes, forming “phagocytic cups” around the apoptotic cells [4,38]. Ultimately, the apoptotic cells are broken down and recycled, thereby promoting inflammation resolution and tissue repair [4,38]. In addition to efferocytosis, MerTK has many other functions related to cardiac dysfunction, including inflammation resolution, oxidative stress production, calcium influx, matrix metalloproteinases (MMPs) activation, and platelet activation and aggregation. First, MerTK is considered as an anti-inflammatory factor that plays a key role in inflammation resolution [39]. It has been shown that MerTK upregulation inhibits the pro-inflammatory NF-κB pathway and induces the secretion of anti-inflammatory cytokines including IL-10 and TGF-β [38,39]. Second, MerTK is activated in response to oxidative stress and promotes macrophage survival by triggering anti-apoptotic pathways and reducing caspase-3 cleavage [40]. Third, MerTK regulates calcium influx that is necessary for the internalization of apoptotic cells. MerTK activation suppresses Ca^2+^/calmodulin-dependent protein kinase II (CaMKII) pathway and inhibits p38 MAPK activity, promoting the synthesis of specialized pro-resolving mediators [41]. Fourth, MMPs are zinc-dependent enzymes that regulate cardiac function by breaking down extracellular matrix (ECM) proteins, playing a crucial role in cardiac remodeling after injury [42]. Recent studies have shown that MerTK regulates the expression of MMP-9 and MMP-7, play important roles in tissue fibrosis formation [43]. Finally, platelet activation and aggregation are critical in cardiac function, serving as the primary response to vascular injury and preventing bleeding [44]. Pathologically, excessive platelet activation and aggregation drive plaque formation, rupture and thrombosis, causing myocardial infarction and subsequent heart failure [44]. MerTK acts as a key mediator in platelet function, supporting platelet aggregation and ensuring stable clot formation [45]. A small-molecule inhibitor of MERTK, UNC2025, has been shown to reduce platelet activation in vitro and prevent thrombus formation in vivo [45]. These MerTK functions well explain our findings of MerTK expression in coronary ECs based on snRNA-seq in human cardiac hypertrophy. As the first barrier between the intravascular and extravascular compartments, ECs act as the initial protective interface against pathogens and leukocyte infiltration [46]. Recent studies by us and others have shown that endothelial MerTK plays a beneficial role in protecting vascular function [[5], [6], [7], [8], [9], [10], [11]]. The increased endothelial MerTK expression in DCM and HCM groups indicates a protective mechanism in response to cardiac injury. In contrast, decreased MerTK expression in macrophages suggests impaired efferocytosis in professional phagocytes and gradually deteriorating condition in cardiac tissues from DCM and HCM groups compared to control groups.

In addition to performing efferocytosis, MerTK has many other functions that are closely associated cardiac dysfunction in the condition of high fat diet, including inflammation resolution, oxidative stress generation, vascular dysfunction and plaque stability [[3], [4], [5], [6], [7], [8], [9], [10], [11]]. MerTK binds to ligands like Gas6 or Protein S, recognizing apoptotic cells and promoting their removal [4]. Timely clearance of apoptotic cells can attenuate pro-inflammatory responses, thereby prevent secondary necrosis and promote tissue repair. Studies in atherosclerosis and aortic aneurysm have shown that MerTK plays an important role in regulating oxidative stress generation, vascular dysfunctions (e.g., endothelial inflammation and SMC phenotypic alterations) and plaque stability [[3], [4], [5], [6], [7], [8], [9], [10], [11]]. Consistently, the current study demonstrates that endothelial MerTK impairment promotes cardiac fibrosis formation, SMC phenotypic switching from contractile state to a synthetic or migratory phenotype in response to high fat diet, and pro-inflammation response. These findings indicate a crucial role of endothelial MerTK in the development of cardiac dysfunction.

High fat diet is linked to diastolic dysfunction that promotes heart failure with preserved ejection fractions in early metrics [1,2]. A recent study demonstrated a relationship between the time course of contractile dysfunction and intrinsic alterations in lipid storage dynamics in response to high fat diet similar to that used in this study [47]. The findings showed that high fat diet leads to impaired glucose tolerance and subsequent cardiac dysfunction [47]. The main mechanisms include reduced lipid storage dynamics and down-regulated adipose triglyceride lipase in the heart [47]. Regarding another mechanism of vascular permeability in cardiac dysfunction, in which the endothelial barrier becomes leaky that represents a hallmark of increased inflammation response in cardiac dysfunction [48]. Vascular permeability is the selective capacity of blood vessels to allow fluid and immune cells to transfer between blood and tissues [49]. Vascular permeability significantly contributes to cardiac dysfunction, including myocardial inflammation, capillary fragility, and impaired diastolic function [48,49]. Recent publications highlighted that endothelial MerTK impairment promotes endothelial dysfunction and phenotypic switching from a contractile to a synthetic state in SMCs, both are the major causes for increased vascular permeability and contribute to cardiac dysfunction [[3], [4], [5], [6], [7], [8], [9], [10], [11]]. This points to a promising study direction for the role of endothelial MerTK in regulating vascular permeability-mediated cardiac dysfunction, such as diastolic dysfunction, decreased cardiac output, and abnormal preserved ejection fraction.

In summary, integrating multi-omics (proteomics, scRNA-seq and snRNA-seq) and the immunostaining validation, along with our unique high fat diet model established in *MerTK*^*flox/flox*^*/Tie2*^*Cre*^ mice, our findings provide compelling evidence that endothelial MerTK impairment represents a novel mechanism in the pathogenesis of cardiac fibrosis and cardiac hypertrophy, contributing to cardiac dysfunction. These novel findings have significant clinical relevance, providing the principle for the restoration of endothelial MerTK to slow down or prevent the development of cardiac dysfunction.

## Data sharing

The authors of this investigation declare that all the data, analytical methods, and study materials are available to the researchers. All the detailed information is available in the Supplemental Data.

## CRediT authorship contribution statement

**Hongye Huang:** Data curation, Formal analysis, Methodology, Validation. **Shijie Liu:** Conceptualization, Data curation, Methodology, Validation. **Jingke Yao:** Data curation, Methodology, Software, Validation. **Xiaoyuan Bai:** Data curation, Methodology, Software, Validation. **Zhicheng Jin:** Validation, Writing – review & editing. **Bingzhong Xue:** Resources, Validation, Writing – review & editing. **Hang Shi:** Software, Validation, Visualization, Writing – review & editing. **Zufeng Ding:** Conceptualization, Data curation, Formal analysis, Funding acquisition, Investigation, Methodology, Project administration, Resources, Software, Supervision, Validation, Visualization, Writing – original draft, Writing – review & editing.

## Declaration of competing interest

The authors declare that they have no conflict of interest.

## Data Availability

Data will be made available on request.
